# Anatomical features decide the atypical seizure manifestation of parahypothalamic hamartomas

**DOI:** 10.3389/fneur.2022.981488

**Published:** 2022-09-12

**Authors:** Chang Liu, Wenhan Hu, Chao Zhang, Zhong Zheng, Xiaoli Yang, Xiu Wang, Jiajie Mo, Zhihao Guo, Xiaoqiu Shao, Kai Zhang

**Affiliations:** ^1^Department of Neurosurgery, Beijing Tiantan Hospital, Capital Medical University, Beijing, China; ^2^Stereotactic and Functional Neurosurgery Laboratory, Beijing Neurosurgical Institute, Capital Medical University, Beijing, China; ^3^Epilepsy Center, Beijing Fengtai Hospital, Beijing, China; ^4^Department of Neurology, Beijing Tiantan Hospital, Capital Medical University, Beijing, China

**Keywords:** hypothalamic hamartoma, stereo-electroencephalography, seizure, semiology, network

## Abstract

**Background:**

The intrahypothalamic phenotype of hypothalamic hamartomas (HH) is associated with epilepsy, and the parahypothalamic phenotype usually leads to central precocious puberty but not neurological comorbidities or seizures. No study has confirmed the pathological role of parahypothalamic hamartomas in epileptogenesis, and the underlying mechanism is yet to be elucidated.

**Objective:**

We aimed to investigate whether parahypothalamic hamartomas are intrinsically epileptogenic and elucidate the underlying pathway of epileptogenesis.

**Methods:**

We reviewed 92 patients with HH-related epilepsy, categorized them by the classification system of Delalande and Fohlen, and further classified Type I (corresponding to parahypothalamic HH) into the following three groups based on the relationship between the lesion and mammillary bodies (MB): entirely invaded (Group 1), partially connected (Group 2), and not connected at all (Group 3). We examined different anatomical features with their relationship to clinical manifestations. Stereoelectroencephalography (SEEG) was implanted in both HH and extra-HH cortices in different groups to identify the epileptogenic zone. Corticocortical evoked potentials (CCEPs) were also used to determine the pathological correlation among different regions to determine the related epileptogenic network.

**Results:**

A total of 13 patients presented with parahypothalamic HH and 10 (76.9%) presented with non-GS only, with late-onset age and normal cognitive development, which is different from classical clinical features. SEEG showed that HH is intrinsically epileptogenic in MB-involved parahypothalamic groups. No statistical difference was found in onset age (*p* = 0.213), and lesions horizontally oriented from the tuber cinereum without connection to MB were not involved in seizure genesis. CCEP indicated a pathological connection among HH, middle cingulate cortex, and insular cortex.

**Conclusion:**

The parahypothalamic HH can also cause epilepsy and is different from classic HH-related seizures, by non-GS only with the late-onset age and normal cognitive development. MB is proven to be related to non-GS by the mamillo-cingulate-cortex pathway.

## Introduction

Hypothalamic hamartomas (HH) are rare, and neurodevelopmental lesions formed from heterotopic neurons and glia within and around the hypothalamus. They arise from the floor of the third ventricle, tuber cinereum (TC), or mammillary bodies (MB) (1). The classical clinical manifestation of HH syndrome includes epilepsy, developmental retardation with behavioral disorders, and central precocious puberty (PP) (2, 3). Of all the seizure types, gelastic seizures (GS) are the distinctive seizure type and the first symptom in virtually all patients with HH (4, 5). Munari et al. (6) reported that ictal discharges associated with GS arise within the HH lesion itself, and many neurophysiological and functional neuroimaging studies have reported that HH is intrinsically epileptogenic (7). Meanwhile, whether other seizure types also arise from HH is debatable, and HH with epilepsy but no history of GS (NGS) is poorly known. In addition, an agreement exists that HH has strong anatomical–clinical correlations in the clinical presentation, and each phenotype is associated with a typical clinical spectrum (8, 9). The intrahypothalamic variant is assumed to be associated with epilepsy with GS and other seizure types and is often pharmacoresistant (2). The parahypothalamic variant usually causes central precocious puberty. However, these patients do not develop severe neurological comorbidities or epilepsy (10–12). Many studies and clinical cases from our center have reported that parahypothalamic hamartomas can also present with epilepsy, even though this is a rare observation (10, 13, 14). So far, no study has confirmed the pathological role of parahypothalamic hamartomas in epileptogenesis and elucidated the underlying mechanism.

In this study, we characterized many patients with parahypothalamic HH with epilepsy, from a clinical, anatomic, and electrophysiological point of view and tried to interpret the results with the present knowledge of the epilepsy manifestation and the relationship with anatomical features to elucidate the underlying mechanism of seizure genesis in parahypothalamic cases.

## Materials and methods

### Patient selection and workup

During May 2015–2022, ninety-two consecutive patients with HH-related epilepsy were retrospectively evaluated. This study was approved by the Institutional Review Boards of Beijing Tiantan Hospital. All the patients diagnosed with HH-related epilepsy at our center underwent a routine workup for presurgical evaluation. High-resolution 3.0-T MRI was obtained of the whole brain, including a 3D T1 sagittal magnetization prepared rapid gradient echo sequence (MPRAGE), T2 axial and coronal sequences of 3-mm thickness, and FLAIR axial, coronal, sagittal sequences of 3-mm thickness, which allowed the visualization in sagittal and coronal planes to better characterize the connection of the hamartoma to the hypothalamus. Interictal [18F] fluorodeoxyglucose (FDG)-PET was also performed and co-registered to the corresponding 3D T1 images using SPM12 (displayed in MRICron http://people.cas.sc.edu/rorden/mricron/index.html) and superimposed on the 3D T1 images with spectrum set at 60–80% transparency (15). A long-term scalp video-EEG recording with 21 channels according to the international 10–20 system was performed for each patient, and at least two habitual seizures were tried to be recorded. After considering the data generated by the abovementioned examinations, all patients underwent a comprehensive evaluation.

### Anatomical classification

**Figure 1:**
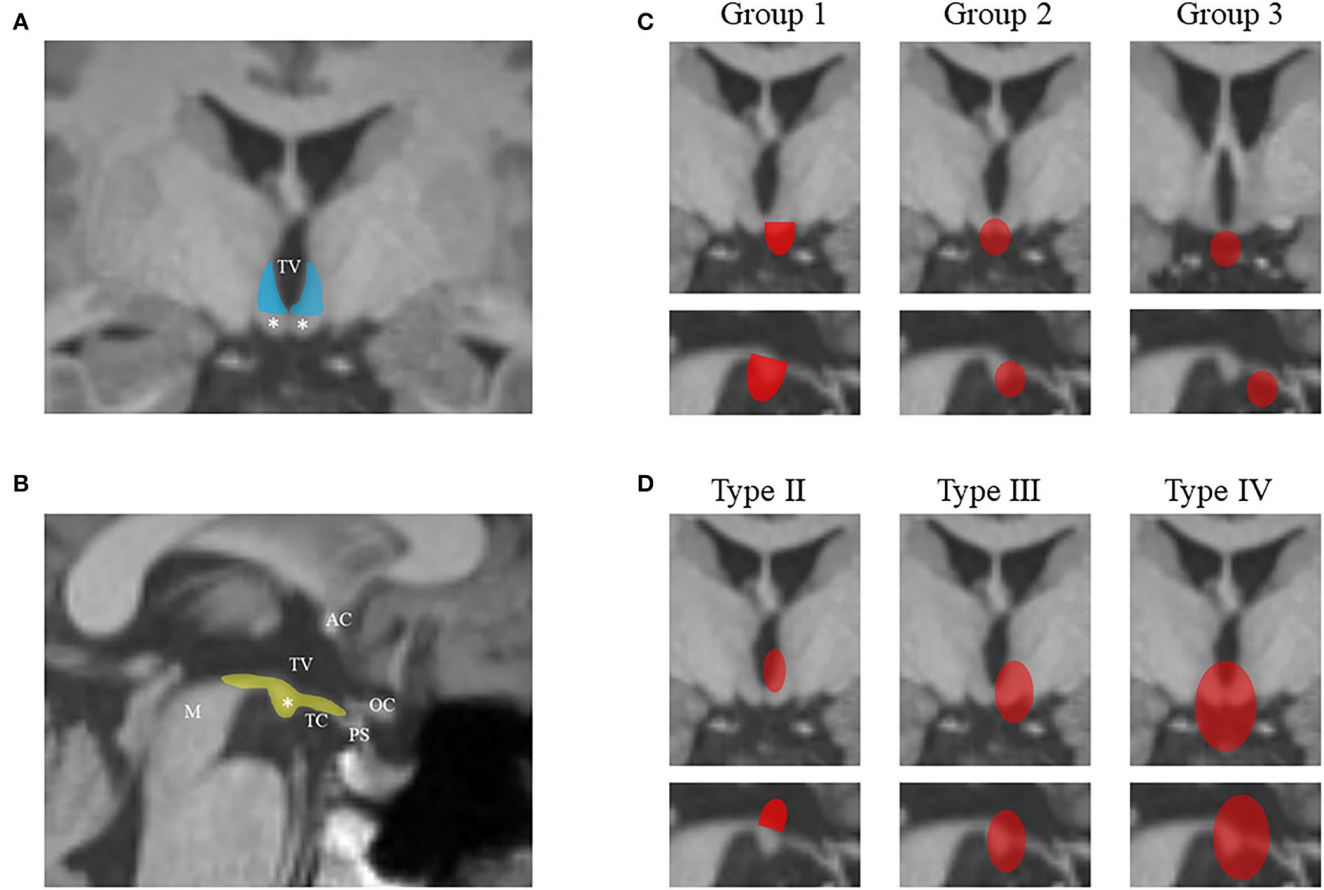
The diagram shows the hypothalamus-related structures from the coronal scan **(A)** and sagittal scan **(B)** of MRI. The lateral hypothalamus from the vertical plane is colored in blue, and the structures on the horizontal plane are colored in yellow. The mammillary bodies are marked by white asterisks. The classification system in this study is illustrated in **(C)** and **(D)** using coronal and sagittal scans. HHs are colored in red. **(C)** The modified classification of Delalande Type I (lesions have a horizontal base of attachment below the normal position of the subventricular floor) based on the relationship between the lesion and MB: entire invasion (Group 1), partial connection (Group 2), and no connection (Group 3). **(D)** Other three types of Delalande classification. Type II lesions have a vertical plane of attachment to the wall of the third ventricle, completely above the subventricular floor. Type III lesions have a plane of attachment that extends both above and below the subventricular floor. Consequently, these lesions have both vertical and horizontal planes of attachment. Type IV lesions were termed “giant” with bilateral insertion. MRI, magnetic resonance imaging; HH, hypothalamic hamartoma; AC, anterior commissure; M, midbrain; OC, optic chiasma; PS, pituitary stalk; TC, tuber cinereum; TV, the third ventricle.

We grouped the study subjects into four types with reference to the classification system of Delalande and Fohlen (16): “horizontally” inserted hamartoma (Type I, corresponding to parahypothalamic HH), “vertically” inserted intraventricular hamartoma (Type II), unilateral “horizontal and vertical” plane of insertion (Type III), and the “giant” lesion with bilateral insertion (Type IV). A modified classification was added for Type I to better determine the relationship between the lesion and MB, by entirely invaded (Group 1), partially connected (Group 2), and not connected at all (Group 3) (Figure 1). For patients who had undergone HH-related surgery earlier, the classification was applied only when there was no deformation of the third ventricle.

### Surgical strategy and invasive recording

After a comprehensive evaluation, patients with short seizure duration and/or less seizure frequency were not considered for surgical treatment, and others were considered for HH-related ablation, including radiofrequency thermocoagulation (RFTC) and magnetic resonance-guided laser interstitial thermal therapy (MRgLITT).

To determine the role of HH in seizure genesis and/or to guide stereotactic ablation, stereoelectroencephalography (SEEG) was designed as per the two strategies:

1 Strategy A: Less than five electrodes were implanted only within the target of HH, mainly for SEEG-guided RFTC by the same recording electrodes. This strategy was applied to the HH cases with a typical history of GS, and the size of the lesion was deemed suitable for RFTC (17).
2 Strategy B: More than five electrodes were implanted in HH and different extra-HH cortical regions to confirm whether HH was the epileptogenic zone (EZ). Criteria for this strategy mainly involved the following 2 situations:
  a Patients who suffered from epilepsy with a HH presentation on neuroimages, but with no history of GS.
  b Patients with a typical history of GS but non-GS were not meaningfully controlled after HH-aiming surgeries.

For all patients, the primary target was the HH, and SEEG electrodes or laser probes were planned to cover the attachment as much as possible in order to make a complete disconnection during ablation. For Strategy B, extra-HH electrode positioning was established in each patient based on the hypotheses about the localization of the EZ according to semiology, scalp EEG, and neuroimaging results.

### SEEG recording and electrical stimulation

Long-term recordings were acquired using intracerebral multiple contact electrodes (8–16 contacts, length: 2 mm, diameter: 0.8 mm, 1.5 mm apart; Beijing HKHS Healthcare Co., Ltd.). The signals were recorded on the NIHON KOHDEN video-EEG monitoring system, sampling at 1,000 or 2,000 Hz to capture several seizures in each patient who underwent SEEG. All seizure episodes recorded by SEEG were visually reviewed by two reviewers and then discussed with a senior neurophysiologist.

For cases with SEEG implantation of Strategy B, direct electrical stimulation was performed with a pulse duration of 300 ms and an intensity of 1–5 mA. In addition, cortico-cortical evoked potentials (CCEP) were also performed. The methodology has been described in detail in a previous study (18).

### Postoperative follow-up

The postoperative outcomes were determined by a clinic visit or *via* telephone. The seizure outcomes were established according to Engel's outcome classification system, and Engel class 1A was considered to be seizure-free after the surgery.

### Statistical analysis

The demographic features were analyzed. The age at epilepsy onset was described as mean, range, and/or standard deviation. An independent sample *t*-test was applied to test the differences among the groups. Statistical tests were performed with the software Statistical Package for the Social Sciences (SPSS) for Windows (version 26). *p* < 0.05 was considered to indicate statistical significance.

## Results

**Figure 2:**
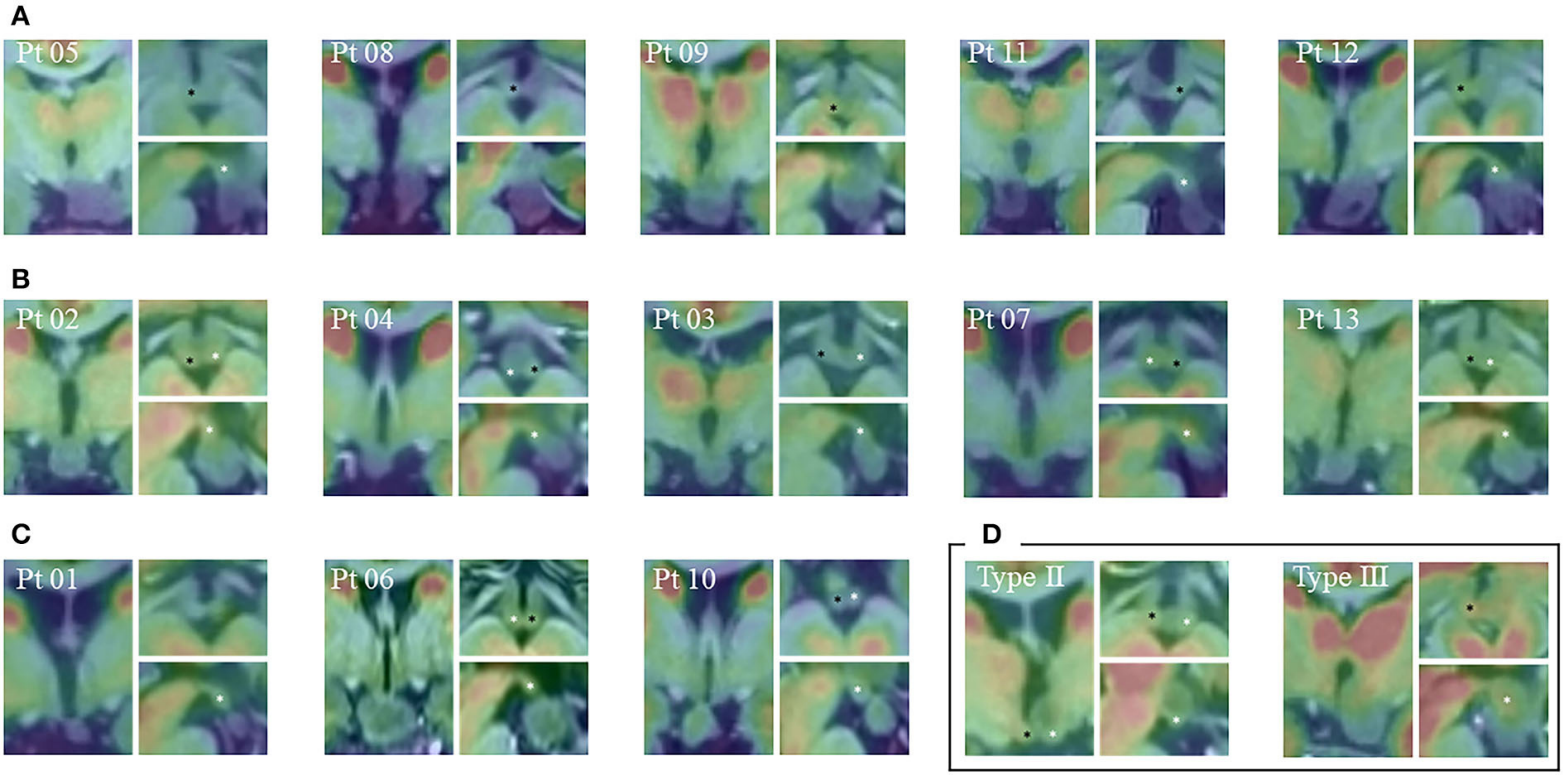
Co-registered images from MRI and PET-CT of each patient by coronal (left), axial (upper right), and sagittal (lower right) scans. Ipsilateral MB and contralateral MB are marked by white and black asterisks, respectively. **(A)** Five patients from Group 1 showed that the hypothalamic connection was established only on one side of the subventricular floor by a sessile attachment, with invaded ipsilateral MB and preserved contralateral MB. **(B)** Five patients from Group 2 showed that HH adheres to both TC and MB outside the third ventricle. In this way, the lesion was bilaterally connected to the MB by its caudal part, and on the coronal plane, narrow attachments were observed on both sides. **(C)** Three patients from Group 3 showed HH horizontally oriented from TC; therefore, the lesion adhered to the subventricular floor only by a pedunculated attachment, being suspended and sparing the MB. **(D)** The relationship between MB and other Delalande types of lesions. MRI, magnetic resonance imaging; PET-CT, positron emission tomography-computed tomography; MB, mammillary bodies; HH, hypothalamic hamartoma; TC, tuber cinereum.

We reviewed a total of 92 epileptic patients with HHs (63 men and 29 women), and the mean age of the patients was 10.5 ±9.1 years (range: 1.8–46 years). A total of 13 patients fulfilled the criterion of Type I (parahypothalamic hamartomas) and were categorized by the modified classification that was previously mentioned (Figure 2). Among the 92 patients, 10 presented with NGS, and all of them belonged to parahypothalamic cases, and three patients of Type I had a history of GS.

In Group 1 (MB entirely invaded), the hypothalamic connection was established only on one side of the subventricular floor by a sessile attachment, and the contralateral MB was preserved. In this group, five patients fulfilled the anatomical criterion, the mean age at seizure onset was 6.2 ± 3.9 years (range 1–11), and the mean duration of epilepsy before the surgical evaluation was 2.1 ± 1.7 years (range 0.5–5). A total of three patients presented with NGS, which included tonic seizure, FIAS, and FBTCS. A total of four patients were treated with HH-aiming ablation (RFTC = 1, LITT = 3), and all of them received seizure freedom with a mean follow-up of 18 months (range: 11–37 months). Patient 8 was implanted with an SEEG plan, and his GS vanished after HH-resection, but FBTCS relapsed. SEEG confirmed that HH was EZ. After LITT, his seizure was present with less frequency till 3 months, which could probably be explained by the “running down” phenomenon (19).

In Group 2 (MB partially connected), HH adhered to both TC and MB outside the third ventricle. In this way, the lesion was bilaterally connected to MB by its caudal part. Therefore, we observed narrow attachments on both sides of the coronal plane. One epileptic-side attachment and interictal discharges and the laterality of hypometabolism were consistent with it. A total of five patients fulfilled the anatomical criterion, the median age at seizure onset was 9.6 ± 4.0 years (range: 5–16), and the mean duration of epilepsy before the surgical evaluation was 5.8 ± 2.9 years (range: 2–10 years). A total of four patients were NGS, and only one patient had a history of GS. However, unlike the classic clinical course, Patient 4 started GS at the age of 5 years, and tonic seizures started at the same time. All these patients underwent SEEG, and two of them were planted with extra-HH regions. One patient did not record the habitual seizures after SEEG monitoring; therefore, subsequent surgery was not considered. The other patient proved HH as EZ and underwent HH-ablation with the other three patients. All four cases received seizure freedom, and the only GS patient (Pt 4) experienced a running-down period of 6 months.

In Group 3 (MB not connected at all), HH was horizontally oriented from TC. Therefore, the lesion adhered to the subventricular floor only by a pedunculated attachment, thereby being suspended and sparing the MB. A total of three patients fulfilled the anatomical criterion, and all of them never suffered from GS, with a very late seizure onset age (16, 30, and 21 years), and showed normal cognitive and physiological development. The mean duration of epilepsy before the surgical evaluation was 5.7 years (range: 1–15). Patient 1 underwent a previous surgery of standard anterior temporal lobectomy for both the seizure and semiology. The results of EEG monitoring showed that the right temporal region was the epileptogenic focus (20). However, the resection of this supposed cortical epileptogenic area failed to control seizures. After 6 years, this patient underwent SEEG-guided RFTC with one electrode placed in the HH. However, ictal discharge did not show low-voltage fast activity, but interictal discharges were recorded. After HH ablation, seizures did not show any improvement by the same seizure frequency and type as preoperative. Patient 10 showed the same preoperative-evaluation results as Patient 1. By this time, an SEEG plan of eight electrodes was implanted on both sides. However, the SEEG results showed that the left hippocampus (HIP) and temporal pole were the seizure onset zone. After an HH-target RFTC, the seizure of this patient did not change at all, with the same seizure frequency and type as preoperative. Patient 6 only had four times of seizures, so she was not considered for surgical treatment after evaluation.

The following three categories of lesions are established for the anatomic configuration of the parahypothalamic HH:

(1) Lesions are horizontally oriented from TC without being connected to MB. They are presented with NGS and are not involved in seizure genesis.
(2) Lesions are simultaneously connected to MB and TC. They always show bilateral attachments to MB with one side being epileptic. HH is the epileptogenic zone, and most patients (80%) presented NGS with a good outcome after HH-aiming ablation.
(3) Lesions that adhere to one side of the subventricular floor showed a sessile attachment to the hypothalamus, with a higher rate of GS compared with Group 2. HH is the EZ, and 60% of the patients presented NGS with a good outcome after HH-aiming ablation.

**Figure 3:**
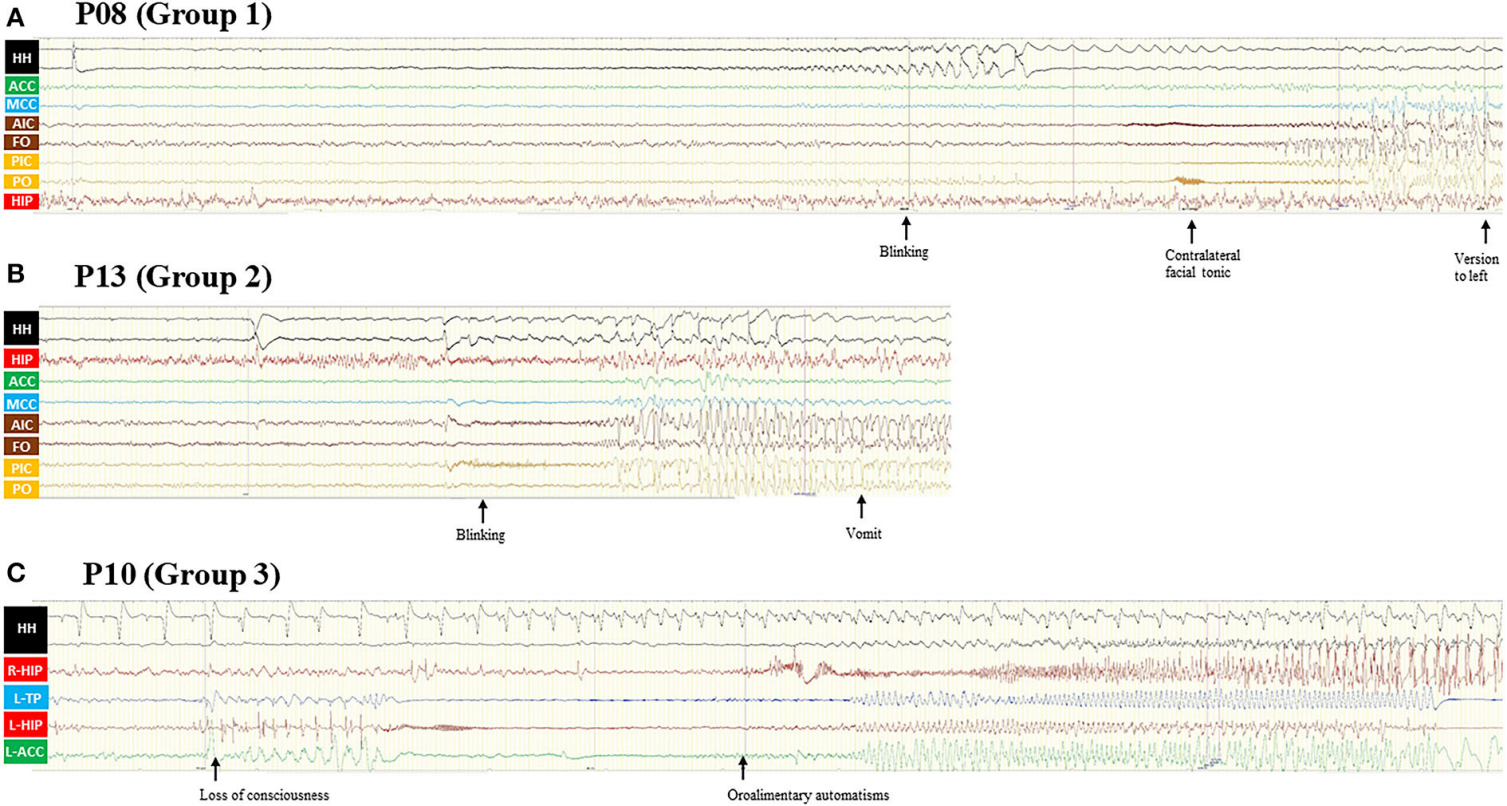
**(A)** Patient 8 was a 12-year-old boy with normal cognitive and physiological development. GS started at the age of 9 years and disappeared after HH-resection 3 years ago, and FBTCS relapsed. Semiology includes eye blinking and a contralateral facial tonic seizure and finally evolving into a bilateral asymmetric tonic seizure. The ictal discharge first appeared in HH and then propagated to the INS, insular operculum, and MCC. **(B)** Patient 13 was a 15-year-old boy with a seizure duration of 5 years. The only seizure type of this patient was eye blinking with awareness, accompanied by flush and vomit sometimes. SEEG recording showed HH onset and then propagated to the INS, insular operculum, and MCC. **(C)** Patient 10 was a 22-year-old male with normal cognitive and physiological development. Seizure presented at the age of 21 years with typical unconscious oroalimentary and hand automatism. SEEG recording showed an onset from the left HIP and temporal pole and then propagated to the ipsilateral ACC, contralateral HIP, and HH. Even though the HH attachment of HH has rhythm changed, the interictal discharge of HH remained nearly the same. GS, gelastic seizures; FBTCS, focal to bilateral tonic-clonic seizures; SEEG, stereo-electroencephalography; HH, hypothalamic hamartoma; ACC, anterior cingulate cortex; MCC, middle cingulate cortex; AIC, anterior insular cortex; PIC, posterior insular cortex; FO, frontal operculum; PO, parietal operculum; HIP, hippocampus; TP, temporal pole; L, left; R, right.

Interictal epileptiform discharges were generally present in the anterior quadrants with spikes localizing over the frontal and/or temporal regions. The ictal EEG can show diffused attenuation without a clear onset zone. Similarly, PET-CT results showed asymmetric cerebral hypometabolism over frontal, temporal, or even parietal lobes. Both of them were usually concordant with the side of attachment of unilateral HH or greater attachment in asymmetric HH. The detailed features of the patients are presented in Table 1, and SEEG results are presented in Figure 3. No statistical difference was found in the onset age between the two MB-involved groups (*p* = 0.213).

**Table 1:** Patient demographics, preoperative work-up, and outcomes.

	**Pt. No**	**Age(y)/ gender**	**Age at onset (y)**	**Seizure duration (y)**	**Seizure types by history and seizure frequency**	**Comorb idity**	**Lesion relationship with hypothalamus**		**EEG IID**	**PET**	**Laterality**	**Previous treatment**	**Surgery**	**Follow-up period (m)**	**Engel outcome**
							**MB**	**Lateral hypothalamus**							
Group 1	5	6/F	5	1	GS daily FIAS weekly		U invaded	Sessile adhered	L: F, T	L: T	L		RFTC	37	IA
	8	12/M	9	3	GS (remission after HH-resection) FBTCS weekly		U invaded	Changed by resection	L: F, T, P	L: T, B: F	L	HH-resection	LITT	11	IA
	9	12/M	11	0.5	FIAS monthly FBTCS monthly		U invaded	Sessile adhered	L: F, T, O	L: T, P	L		—	—	—
	11	6/M	1	5	Tonic seizure daily GTCS monthly		U invaded	Sessile adhered	B: F, T, P, O	R: F, T, P	R	HH-resection	LITT	12	IA(RD)
	12	6/F	5	1	FBTCS weekly		U invaded	Sessile adhered	L: F, T, O	L: F, T	L	HH-RFTC	LITT	12	IA
Group 2	2	18/F	8	10	FIAS weekly +/–BATS		B connected	Not invaded	L: T	L: T	L		RFTC	57	IA
	3	24/F	16	8	FIAS monthly		B connected	Not invaded	R: F, T	R: F, T	R		RFTC	50	IA
	4	9/M	5	4	GS daily Tonic seizure daily	Cognitive impairment	B connected	Not invaded	B: F, T	L: T, B: F, P	L		RFTC	31	IA(RD)
	7	11/F	9	2	FIAS weekly FBTCS monthly	Precocious puberty	B connected	Not invaded	R: F, T	R: F, B: T, P	R		—	—	—
	13	15/M	10	5	FAS daily		B connected	Not invaded	L: T	L: F, T	L		LITT	10	IA
Group 3	1	31/F	16	15	FIAS daily		Not connected	Not invaded	R: T	L: T	B	R -ATL	RFTC	71	No change
	6	30/F	30	1	GTCS four times		Not connected	Not invaded	R: F, T	R: T	R		—	—	—
	10	22/M	21	1	FIAS daily		Not connected	Not invaded	B: T	L: T, F, P	L		RFTC	13	No change

F, frontal; T, temporal; P, parietal; O, occipital; B, bilateral; U, unilateral; L, left; R, right; ATL, anterior temporallobectomy; RD, running-down; FIAS, focal impaired awareness seizure; GTCS, generalized tonic-clonic seizure; FBTCS, focal to bilateral tonic-clonic seizure; FAS, focal aware seizure; BATS, bilateral asymmetric tonic seizure.

**Figure 4:**
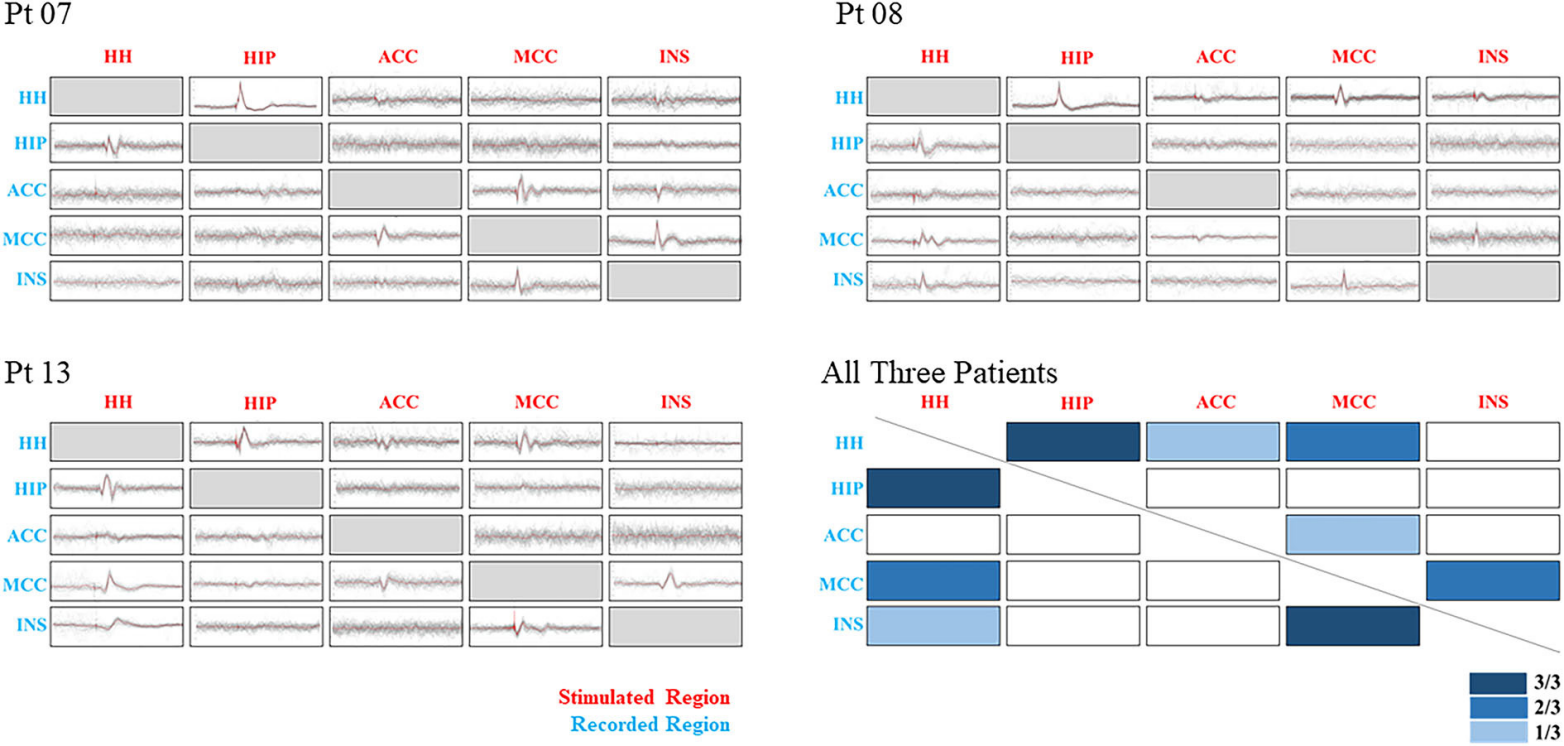
Showing CCEP results of three patients and their combining results. Five regions were selected to be shown in different ranks by stimulated and recorded conditions. The *x*-axis stands for the stimulated regions, and the *y*-axis stands for the recorded regions. Three degrees of the color bar were used to show the different combining results, with a darker bar corresponded to a higher positive rate. Pathological relationships were observed between HH-HIP, MCC-INS, and HH-MCC. CCEP, cortico-cortical evoked potentials; HH, hypothalamic hamartoma; HIP, hippocampus; ACC, anterior cingulate cortex; MCC, middle cingulate cortex; INS, insular cortex.

Stimulation of different regions of gray matter can produce different CCEPs under normal baseline conditions, which requires consideration of the baseline response while determining the effects of epilepsy (18). A total of five cortical and subcortical regions of three patients were selected for further analysis by consistency, which included HH, HIP, anterior cingulate cortex (ACC), middle cingulate cortex (MCC), and insular cortex (INS). Results showed a bidirectional connection from HH to HIP and a unidirectional connection from MCC to INS in all three cases. Other pathological connections included HH-MCC, INS to MCC in two cases, and HH to INS only in one case (Figure 4).

## Discussion

To the best of our knowledge, no study has elucidated the pathogenetic role underlying HH in patients with epilepsy without GS. In this study, we proved that parahypothalamic HH can also cause epilepsy by SEEG, and this type of epilepsy is very different from classic HH-related seizures. NGS is in most cases with a very late-onset age and normal cognition. MB is speculated to be related to non-GS in patients with HH by the MB-cingulate-cortex pathway.

Hypothalamic hamartomas are developmental malformations that involve the small hypothalamic area located between the infundibular stalk and MB. Pedunculate hamartomas are characterized by adhering to the inferior surface of the hypothalamus, often with a thin stalk that arises from the region of TC and MB (corresponding to Groups 2 and 3), whereas sessile hamartomas are characterized by broad-based attachment to the hypothalamus (12, 21). The latter can be further divided into many variants based on the different sites of attachment of the lesion, which included parahypothalamic adhesions (corresponding to Group 1), intrahypothalamic adhesions (corresponding to Delalande Type II), and combined unilaterally (Delalande Type III) or bilaterally (Delalande Type IV) (Figure 1). Many subsequent classifications are more detailed about the relation to size, width, location of attachment, and distortion of the hypothalamus (22, 23). Some of the classifications were designed to select an appropriate operative surgical strategy, and some of these classifications correlated the physical characteristics of HH with clinical features (23, 24).

Hypothalamic hamartomas have strong anatomical–clinical correlations in the clinical presentation, and each phenotype is associated with a typical clinical spectrum (8, 11). Different from intrahypothalamic HH, the epileptic features of parahypothalamic HH are not sufficiently studied, and most studies hypothesize that this variant does not develop epilepsy (10–12). The results of many previous studies are consistent with our results. Mullatti et al. (25) reported 19 patients with epileptic HHs, which included 5 “para-hypothalamic” cases. All three pedunculated cases presented with NGS, which included complex partial seizures, drop attacks, atypical absence, and tonic seizures. Li et al. (26) reported 214 surgically resected HHs and classified them as “extra-ventricular” HH, which corresponded to Groups 2 and 3 in this study. For the lesions with a narrow attachment connected to the outside floor of the third ventricle, only 3.9% (3/77) cases had a history of GS, whereas for the lesions with a broad attachment, 26.9% (7/26) had a history of GS (26). Scolly et al. (13) reported one parahypothalamic case with temporal semiology that started at the age of 15 years and proved that the lesion cannot be obligatorily involved in spontaneous seizures by SEEG. Sone et al. (14) reported one pedunculated HH presented with temporal epilepsy from the age of 21 years without PP or intellectual impairment. The parahypothalamic lesion was confirmed to be epileptic by intracranial EEG and successful treatment by thermocoagulation (14). Kameyama et al. (27) reported six parahypothalamic cases from a total of 100 patients, and all the attachments of the lesions were unilateral and had a significantly higher prevalence of PP.

All these results from previous studies and our cohort suggest that parahypothalamic hamartomas play a very specific role in the pathophysiology of seizure genesis. Different epileptogenic networks based on related anatomic features and pathways should be concerned as a reasonable explanation. An agreement exists that hamartomas associated with epilepsy have a sessile displacing attachment to the hypothalamus, whereas those associated with precocious puberty alone are pedunculated, which implied barely distortion within the third ventricle (28). As a result, many studies separately investigated the role of different hypothalamic areas in epilepsy (9). Each group of Kahane et al. (29) and Kameyama et al. (7) proposed two different pathways that included different structures. Kahane et al. proposed speculative pathophysiology wherein the mamillo-thalamo-cingulate tract serves as a relay of HH discharges toward the cortex, the excitability of which would then progressively increase, leading to cortical interictal epileptiform abnormalities and then to seizures of cortical origin (29). Kameyama et al. reported that a thalamo-ponto-cerebellar circuit plays an important role in the stereotypical and automatic symptomatogenesis of GS; however, no positive results were found in the mammillo-thalamo-cingulate pathway (7). To summarize, Kahane et al. suggested that seizures arising from the extra-HH cortex can be related to the MB-cingulate pathway, and Kameyama et al. suggested that GS is related to subcortical structures, including the thalamus and brainstem sparing MB. These findings are consistent with our speculation.

Compared with intrahypothalamic HH, parahypothalamic HH has very different anatomical features. The vertical plane of the third ventricle included lateral hypothalamic nuclei and fornix, and other important tracts were rarely affected. In this way, the structures from the horizontal plane of the third ventricle can be better investigated, including MB, TC, and infundibulum. Since the infundibulum was thought to be associated with the clinical manifestation of PP (8, 21), MB and TC were proposed to be the critical structures leading to epileptic seizures in parahypothalamic HH. The pathological role of TC is not completely known because in two cases from Group 3 with TC-connection only, HH was not involved in seizure genesis even though interictal discharges were recorded. Thus, by excluding lateral hypothalamus involvement, we can investigate the role of MB in seizure genesis more clearly (Table 2). In our cohort, we found that parahypothalamic-HH epilepsy can manifest a spectrum of conditions with seizures by various degrees of MB connectivity. When the lesion originated from the region ventral to MB, there will be a connection between them, and the epileptic HH will lead to only nongelastic seizures in most cases. Correspondingly, intrahypothalamic HH with MB and lateral hypothalamus involved simultaneously did not lead to nongelastic seizures alone in our cohort. Moreover, HH was not involved in seizure genesis at all when it was not connected to MB. Even though multiple additional seizure types developing during the clinical course were thought to be related to a process of secondary epileptogenesis (30) and the concept of “hypothalamic plus” epilepsy was introduced to explain an epileptogenic network extended to a remote cortical region, which becomes an independent “extrahypothalamic” epileptogenic zone (19), we do not consider cases of Group 3 belong to the same situation. Secondary epileptogenesis should occur in the cases suffered from multiple seizure types, and at least one kind of seizure should be confirmed to be raised from the HH.

**Table 2:** Comparison of parahypothalamic HH and intrahypothalamic HH.

	**Parahypothalamic HH**			**Intrahypothalamic HH**
	**Group 1**	**Group 2**	**Group 3**	
Corresponding classification	Regis IV; Li II	Li I	Regis V	Delalande II–IV, Regis I–III, Li III–IV
Relationship with MB	Not connected	Bilateral connected	Unilateral invaded	Unilateral or bilateral invaded
Change of the third ventricle	N	N	N	Y
Precocious puberty	0/5	1/5	0/3	—
Cognitive disturbance	0/5	1/5	0/3	Y
Gelastic seizures	2/5	1/5	0/3	GS and other seizure types
EZ confirmed by SEEG	HH onset	HH onset	extra-HH onset	HH onset
Seizure freedom after HH ablation	4/4	4/4	0/2	—

HH, hypothalamic hamartoma; GS, gelastic seizures; MB, mammillary bodies; SEEG, Stereo-electroencephalography; EZ, epileptogenic zone.

To summarize, we speculated two different pathways, the horizontal and vertical, leading to different seizure types. The horizontal pathway included MB, and HH may evolve into non-GS seizures due to the mamillo-cingulate-cortex pathway involving different extra-HH cortices. The vertical pathway included the lateral hypothalamus and related subcortical structures such as the tegmentum and mediodorsal nucleus of the thalamus, which might be correlated with GS genesis. For the intrahypothalamic HH, the attachment affects the structures involved in the horizontal and vertical pathways simultaneously; therefore, both GS and non-GS will occur in a classic clinical course. Therefore, the lateralization of clinical seizure characteristics and EEG are generally ipsilateral to the side of the HH attachment (31). We speculated that the horizontal pathway included MB, MCC, and INS based on the CCEP results. Additionally, some clinical features also supported the conclusion regarding these two pathways. In this study, seizures arose from parahypothalamic HH *via* MB showing late onset age and multiple seizure types, similar to secondary epileptic progression in patients with typical GS. HH seizures usually occur in <1 year in the case of GS and between the ages of 2 and 7 years in the case of additional focal seizure types (11), and seizures associated with HH are age-dependent (32). We speculated that pathological projections from MB might not be as easily triggered as the GS pathway. As a result, non-GS, including NGS, can occur several years later than GS with less seizure frequency, and in some cases, its partial connection with MB makes it even harder (such as Group 2); therefore, seizures arising from the ventral part of MB are rare. Additionally, most parahypothalamic HH may not affect the third ventricle; thus, the hypothalamic projections from the lateral hypothalamus are not involved in most cases; as a result, parahypothalamic HH is an occasional finding and always presents only endocrinologic disturbances when the infundibular stalk is affected.

We should make it clear that GS and non-GS should not be separated only by these two pathways. Even though we proved that MB might be related to non-GS, we could not prove that the lateral hypothalamus is related only to GS. For example, the fornix also belongs to the lateral hypothalamus and is part of Papez's circuit similar to MB, let alone other anatomic and functional connections between the thalamus, limbic circuitry, and cortices (33). We believe that the exact mechanisms underlying the development of these seizures and related epileptogenic networks should be even more complex, and one specific structure cannot be related to only one type of seizure or lobe, even though an anatomical-related propensity exists.

Even though previous studies speculated that MB might be related to GS (34), most cases from their cohorts have a sessile attachment to the third ventricle. If lesions seated in a region rostral to the MB without the third ventricle distortion are considered, the present results are more convincing. By proving that parahypothalamic HH could also lead to seizures and always atypical types with late-onset age and normal cognition, this study showed a direct relation between MB and non-GS, and hypothalamic structures on the vertical plane might be related to typical GS with early onset age and neurocognitive/behavioral sequelae. Further studies are required to investigate this anatomical correlation in depth.

## Data availability statement

The original contributions presented in the study are included in the article/supplementary material, further inquiries can be directed to the corresponding author/s.

## Ethics statement

The studies involving human participants were reviewed and approved by Institutional Review Boards of Beijing Tiantan Hospital. Written informed consent to participate in this study was provided by the participants' legal guardian/next of kin. Written informed consent was obtained from the individual(s), and minor(s)' legal guardian/next of kin, for the publication of any potentially identifiable images or data included in this article.

## Author contributions

CL: acquisition of data, statistical analysis, and drafting of the manuscript. WH, CZ, XW, and XS: acquisition and interpretation of data and revising the manuscript for intellectual content. ZZ, XY, and ZG: acquisition and analysis of data. JM: interpretation of data and statistical analysis. KZ: study design, study supervision, and final revision of the manuscript for intellectual content. All authors contributed to the article and approved the submitted version.

## Funding

This project was supported by the Capital's Funds for Health Improvement and Research (2022-1-1071 and 2020-2-1076), the National Natural Science Foundation of China (82071457), and the National Key R&D Program of China (2021YFC2401201).

## Conflict of interest

The authors declare that the research was conducted in the absence of any commercial or financial relationships that could be construed as a potential conflict of interest.

## Publisher's note

All claims expressed in this article are solely those of the authors and do not necessarily represent those of their affiliated organizations, or those of the publisher, the editors and the reviewers. Any product that may be evaluated in this article, or claim that may be made by its manufacturer, is not guaranteed or endorsed by the publisher.

## References

[1] Alomari SO Houshiemy MNE Bsat S Moussalem CK Allouh M Omeis IA. Hypothalamic hamartomas: a comprehensive review of the literature - Part 1: Neurobiological features, clinical presentations and advancements in diagnostic tools. Clin Neurol Neurosurg. (2020) 197:106076. 10.1016/j.clineuro.2020.106076 32717559

[2] Kerrigan JF Parsons A Tsang C Simeone K Coons S Wu J. Hypothalamic hamartoma: neuropathology and epileptogenesis. Epilepsia. (2017) 58 Suppl 2:22–31. 10.1111/epi.13752 28591478

[3] Freeman JL Wellard RM Kean MJ Rosenfeld JV Jackson GD Berkovic SF. Imaging and spectroscopic study of epileptogenic hypothalamic hamartomas: analysis of 72 cases. Am J Neuroradiol. (2004) 25:450–62. 15037472 PMC8158567

[4] Striano S Striano P. Clinical features and evolution of the gelastic seizures-hypothalamic hamartoma syndrome. Epilepsia. (2017) 58(Suppl. 2):12–5. 10.1111/epi.13753 28591476

[5] Striano S Striano P Coppola A Romanelli P. The syndrome gelastic seizures-hypothalamic hamartoma: severe, potentially reversible encephalopathy. Epilepsia. (2009) 50 (Suppl. 5):62–5. 10.1111/j.1528-1167.2009.02125.x 19469851

[6] Munari C Kahane P Francione S Hoffmann D Tassi L Cusmai R. Role of the hypothalamic hamartoma in the genesis of gelastic fits (a video-stereo-EEG study). Electroencephalogr Clin Neurophysiol. (1995) 95:154–60. 10.1016/0013-4694(95)00063-5 7555906

[7] Kameyama S Masuda H Murakami H. Ictogenesis and symptomatogenesis of gelastic seizures in hypothalamic hamartomas: an ictal SPECT study. Epilepsia. (2010) 51:2270–9. 10.1111/j.1528-1167.2010.02739.x 20887368

[8] Harrison VS Oatman O Kerrigan JF. Hypothalamic hamartoma with epilepsy: review of endocrine comorbidity. Epilepsia. (2017) 58(Suppl. 2):50–9. 10.1111/epi.13756 28591479 PMC5533614

[9] Hamdi H Ferrante P Spatola G Clawson W McGonigal A Daquin G. Epileptic hypothalamic hamartomas impact of topography on clinical presentation and radiosurgical outcome. Epilepsy Res. (2021) 173:106624. 10.1016/j.eplepsyres.2021.106624 33839515

[10] Chan YM Fenoglio-Simeone KA Paraschos S Muhammad L Troester MM Ng YT. Central precocious puberty due to hypothalamic hamartomas correlates with anatomic features but not with expression of GnRH, TGFalpha, or KISS1. Horm Res Paediatr. (2010) 73:312–9. 10.1159/000308162 20389100 PMC2868525

[11] Cohen NT Cross JH Arzimanoglou A Berkovic SF Kerrigan JF Miller IP. Hypothalamic hamartomas: evolving understanding and management. Neurology. (2021) 97:864–73. 10.1212/WNL.0000000000012773 34607926 PMC8610628

[12] Arita K IF Kurisu K Sumida M Harada K Uozumi T Monden S. The relationship between magnetic resonance imaging findings and clinical manifestations of hypothalamic hamartoma. J Neurosurg. (1999) 91:212–20. 10.3171/jns.1999.91.2.0212 10433309

[13] Scholly J Staack AM Kahane P Scavarda D Regis J Hirsch E. Hypothalamic hamartoma: epileptogenesis beyond the lesion? Epilepsia. (2017) 58(Suppl. 2):32–40. 10.1111/epi.13755 28591482

[14] Sone D Kaido T Watanabe M Murata Y Taniguchi G Otsuki T. Adult-onset refractory epilepsy with hypothalamic hamartoma and no gelastic seizures successfully treated by stereotactic thermocoagulation: a case report. Seizure. (2016) 37:32–4. 10.1016/j.seizure.2016.02.013 26975044

[15] Hu WH Wang X Liu LN Shao XQ Zhang K Ma YS. Multimodality image post-processing in detection of extratemporal MRI-negative cortical dysplasia. Front Neurol. (2018) 9:450. 10.3389/fneur.2018.00450 29963006 PMC6010529

[16] Delalande O Fohlen M. Disconnecting surgical treatment of hypothalamic hamartoma in children and adults with refractory epilepsy and proposal of a new classification. Neurol Med Chir. (2003) 43:61–8. 10.2176/nmc.43.61 12627881

[17] Liu C Zheng Z Shao XQ Li CD Yang XL Zhang C. Stereoelectroencephalography-guided radiofrequency thermocoagulation for hypothalamic hamartoma: electroclinical patterns and the relationship with surgical prognosis. Epilepsy Behav. (2021) 118:107957. 10.1016/j.yebeh.2021.107957 33872942

[18] Guo ZH Zhao BT Toprani S Hu WH Zhang C Wang X. Epileptogenic network of focal epilepsies mapped with cortico-cortical evoked potentials. Clin Neurophysiol. (2020) 131:2657–66. 10.1016/j.clinph.2020.08.012 32957038

[19] Scholly J Valenti MP Staack AM Strobl K Bast T Kehrli P. Hypothalamic hamartoma: is the epileptogenic zone always hypothalamic? Arguments for independent (third stage) secondary epileptogenesis. Epilepsia. (2013) 54(Suppl. 9):123–8. 10.1111/epi.12456 24328885

[20] Yang AC Zhang K Zhang JG Liu HG Chen N Ge M. Temporal lobe epilepsy with hypothalamic hamartoma: a rare case. Chin Med J. (2011) 124:1114–7. 10.3760/cma.j.issn.0366-6999.2011.07.031 21542979

[21] Boyko O CJ Oakes W Burger P. Hamartomas of the tuber cinereum: CT, MR, and pathologic findings. Am J Neuroradiol. (1991) 12:309–14. 1902033 PMC8331418

[22] Valdueza JM CL Dammann O Bentele K Vortmeyer A Saeger W Padberg B. Hypothalamic hamartomas: with special reference to gelastic epilepsy and surgery. Neurosurgery. (1994) 34:949–58. 10.1227/00006123-199406000-00001 8084405

[23] Regis J Scavarda D Tamura M Nagayi M Villeneuve N Bartolomei F. Epilepsy related to hypothalamic hamartomas: surgical management with special reference to gamma knife surgery. Childs Nerv Syst. (2006) 22:881–95. 10.1007/s00381-006-0139-y 16807727

[24] Regis J Helen Cross J Kerrigan JF. Achieving a cure for hypothalamic hamartomas: a Sisyphean quest? Epilepsia. (2017) 58(Suppl. 2):7–11. 10.1111/epi.13773 28591481

[25] Mullatti N Selway R Nashef L Elwes R Honavar M Chandler C. The clinical spectrum of epilepsy in children and adults with hypothalamic hamartoma. Epilepsia. (2003) 44:1310–9. 10.1046/j.1528-1157.2003.04103.x 14510825

[26] Li CD Tang J Jia G Ma ZY Zhang YQ. Classification of hypothalamic hamartoma and prognostic factors for surgical outcome. Acta Neurol Scand. (2014) 130:18–26. 10.1111/ane.12209 24382157

[27] Kameyama S Shirozu H Masuda H Ito Y Sonoda M Akazawa K. MRI-guided stereotactic radiofrequency thermocoagulation for 100 hypothalamic hamartomas. J Neurosurg. (2016) 124:1503–12. 10.3171/2015.4.JNS1582 26587652

[28] Debeneix C BM Trivin C Sainte-Rose C Brauner R. Hypothalamic hamartoma: comparison of clinical presentation and magnetic resonance images. Horm Res. (2001) 56:12–8. 10.1159/000048084 11815722

[29] Kahane PRP Hoffmann D Minotti L Benabid AL. From hypothalamic hamartoma to cortex: what can be learnt from depth recordings and stimulation? Epileptic Disord. (2003) 5:205–17. 14975789

[30] Striano S Santulli L Ianniciello M Ferretti M Romanelli P Striano P. The gelastic seizures-hypothalamic hamartoma syndrome: facts, hypotheses, and perspectives. Epilep Behav. (2012) 24:7–13. 10.1016/j.yebeh.2012.02.013 22503469

[31] Harvey AS Freeman JL. Epilepsy in hypothalamic hamartoma: clinical and EEG features. Semin Pediatr Neurol. (2007) 14:60–4. 10.1016/j.spen.2007.03.003 17544948

[32] Oehl B Brandt A Fauser S Bast T Trippel M Schulze-Bonhage A. Semiologic aspects of epileptic seizures in 31 patients with hypothalamic hamartoma. Epilepsia. (2010) 51:2116–23. 10.1111/j.1528-1167.2010.02686.x 20738381

[33] Freeman JL HA Rosenfeld JV Wrennall JA Bailey CA Berkovic SF. Generalized epilepsy in hypothalamic hamartoma. Evolution and postoperative resolution. Neurology. (2003) 60:762–7. 10.1212/01.WNL.0000049457.05670.7D 12629230

[34] Parvizi J Le S Foster BL Bourgeois B Riviello JJ Prenger E. Gelastic epilepsy and hypothalamic hamartomas: neuroanatomical analysis of brain lesions in 100 patients. Brain. (2011) 134(Pt 10):2960–8. 10.1093/brain/awr235 21975589

